# Marriages of Consanguinity

**Published:** 1862-06

**Authors:** 


### MARRIAGES OF CONSANGUINITY.

Dr. Devay, Professor of Clinical Medicine at the Medical School of Lyons, has just published an interesting work on the disastrous effects of marriages among relations. He shows that in fixing certain prohibited degrees of consanguinity, the Church in point of fact was only favoring the observance of one of the most important laws of nature, the infringement of which is punished with inevitable degeneracy. Unions within the limits of consanguinity are hurtful not only to the human race, but also to animals. It is true that such unions among the latter are promoted by the breeder for profit's sake—the Disley and Durham oxen, so admirable in the eyes of the breeder, are instances of this; but sterility is the usual consequence of the practice. In the human race two circumstances have contributed to favor marriages among relations—the first occurs when a small population is pent up in some remote hamlet not easily accessible. The second case is that of families desirous of maintaining their rank in society, or preventing the dispersion of their fortune by marrying within their own circle. Dr. Devay states that out of 121 marriages of this kind observed by him, 22 were barren. Only 4 of the number were marriages between uncles and grand-nieces—the others were between cousins or the issues of cousins. When sterility does not occur, the issue is diseased, or afflicted with blindness or deafness; also in many cases afflicted with irregularity of conformation. Of all these irregularities, polydactylism, or multiplicity of fingers, is the most frequent. Dr. Devay has observed this in 17 out of the 121 cases above mentioned. He states that in a certain secluded spot, where the inhabitants had no communication with other populations, polydactylism had become quite endemic, and that this strange anomaly disappeared some time after a new road had been cut through the place.—*Paris Correspondence of British American Medical Journal.*

